# Supplementary material for: Photochemical Synthesis of Noble Metal Nanoparticles: Influence of Metal Salt Concentration on Size and Distribution
Source: Int J Mol Sci. 2023 Sep 13;24(18):14018. doi: 10.3390/ijms241814018 (PMC10530956; doi:10.3390/ijms241814018)
Supplement: Supplementary file 1 [file ijms-24-14018-s001.zip › ijms-2535036-supplementary.pdf]

Investigation of the Synthesis and Mechanism of Gold and Silver Nanoparticles Using New One-Component Photoinitiators

Shahad M. Aldebasi [1], Haja Tar [1,*], Abrar S. Alnafisah [1], Lotfi Beji [2], Noura Kouki [1], Fabrice Morlet-Savary [3], Fahad M. Alminderej [1], Lotfi M. Aroua [1] and Jacques Lalevee [3]

[1] Department of Chemistry, College of Science, Qassim University, Buraidah 51452, Saudi Arabia
[2] Department of Physics, College of Sciences and Arts at ArRass, Qassim University, Buraidah 51452, Saudi Arabia.
[3] CNRS, IS2M UMR 7361, Université de Haute-Alsace, F-68100 Mulhouse, France
* Correspondence: h.tar@qu.edu.sa

1. *Synthesis of photoinitiator B*

1.2.Experimental

FT-IR spectra were achieved using Agilent FT-IR Spectrometer Cary 600 with a microscope in the range of 400-4000 cm^{-1}. The ^{1}H and ^{13}C NMR spectra were recorded on a 400 MHz NMR spectrometer Bruker Avance at 400 and 100 MHz, respectively. All spectra were obtained using $CDCl_3$ as a solvent and referenced to TMS. Data are reported as follows: chemical shift, integration, multiplicity (s =singlet, d = doublet, t = triplet, q = quartet, and m = multiplet). Chemical shifts of ^{13}C NMR spectra are reported in ppm from $CDCl_3$. The melting points were measured in open capillary tubes using The Stuart SMP30 Apparatus. Analytical TLC was performed using Silica Gel 60 F254 plates (Sigma 40–60 μm). The developed chromatogram was visualized under a UV lamp (254 nm). All commercially available reagents were purchased from Sigma-Aldrich and used without further purification. All reactions were carried out using oven-dried glassware unless otherwise stated.

1.2. Preparation of 2,2′-(1,2-phenylenebis(azanylylidene))bis(methanylylidene))diphenol (P1) [1]

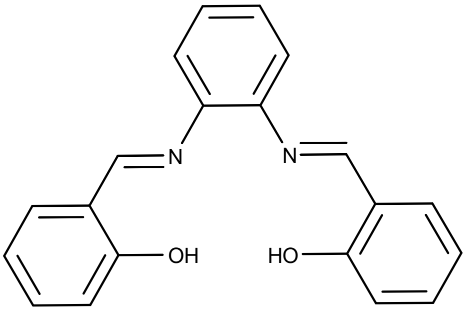

vigorously stirred solution of 1,2-phenylenediamine (2.16 g, 20 mmol) and added dropwise solution of salicylaldehyde (2.44, 20 mmol) in anhydrous ethanol (10 mL) then the mixture was stirred for 6 h. The resulting solution was evaporated in a vacuum to remove the solvent until a solid precipitated. The product was filtered and washed with ethanol, and dried in a vacuum to give a yellow solid, yielding 85%, ^{1}H NMR (400 MHz, $CDCl_3$) δ: 13.09 (s, 2H, OH,), 8.65 (s, 2H, CH=N), 7.44-7.34 (m, 6H, Harom), 7.28-7.23 (m, 2H, Harom), 7.08 (d, 2H, *J*= 8.4Hz, Harom), 6.98-6.93 (t, 2H, *J*= 8.0Hz, Harom); ^{13}C NMR (400 MHz, $CDCl_3$) δ_C: 163.74 (2c, C-OH,), 161.37 (2c, C=N), 142.58 (2c), 133.40 (2c), 132.37 (2c), 127.74 (2c), 119.74 (2c), 119.25(2c), 119.01(2c), 117.58(2c).

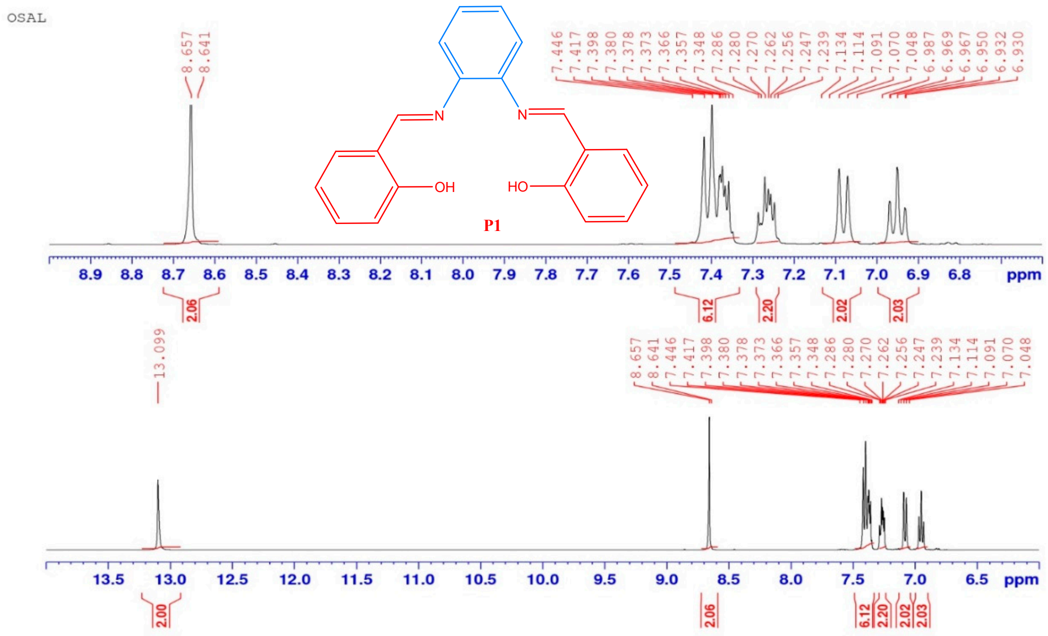

Figure S1. ^{1}H NMR spectra of the photoinitiator B.

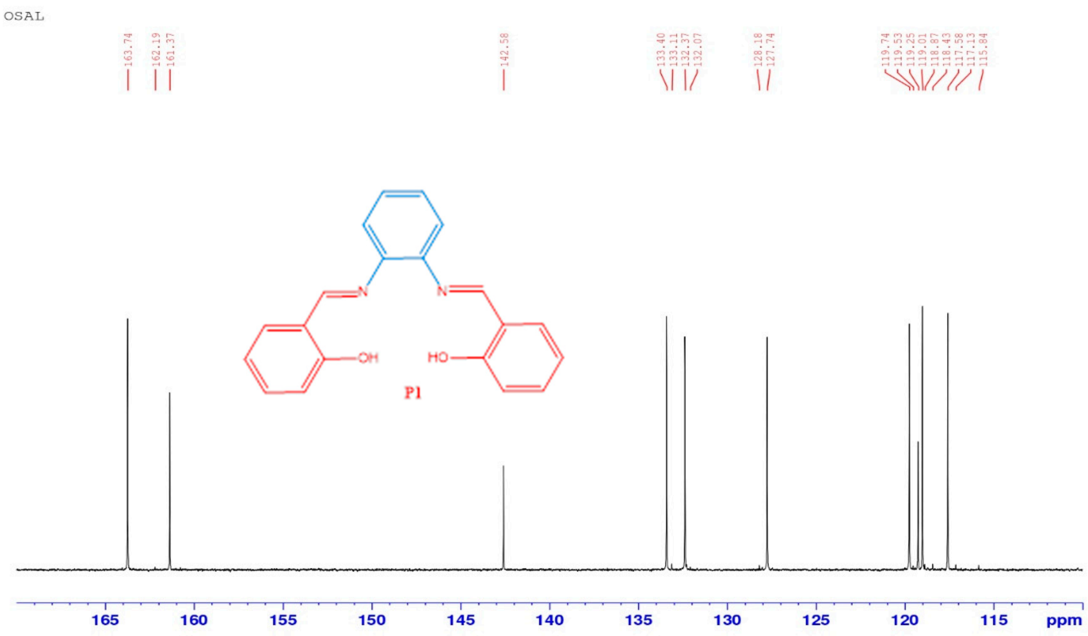

Figure S2. ^{13}C NMR spectra of the photoinitiator B.

2. *Fluorescence experiments of the photoinitiators*

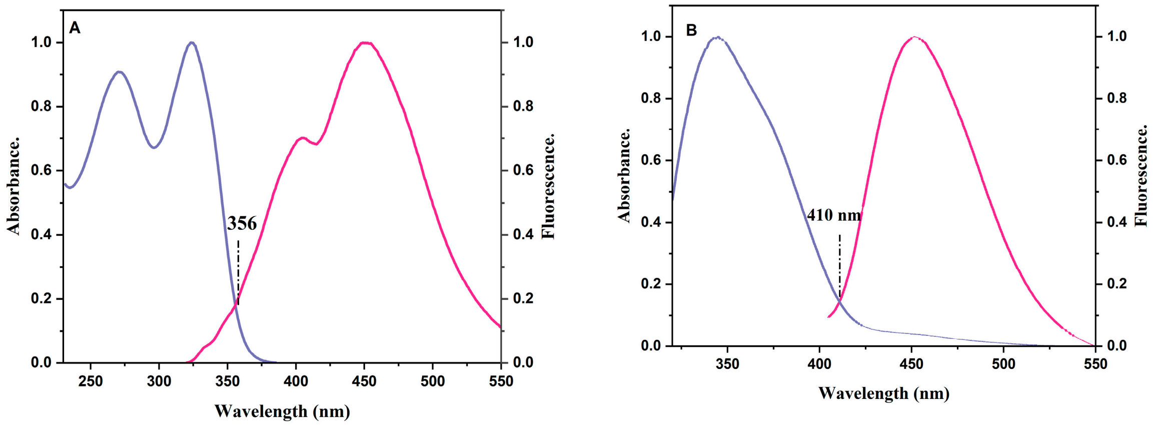

Figure S3. Normalized fluorescence excitation and emission of photoinitiator **A** and B in methanol solution at room temperature.

3. *Photoproduction of Au Nanoparticles*

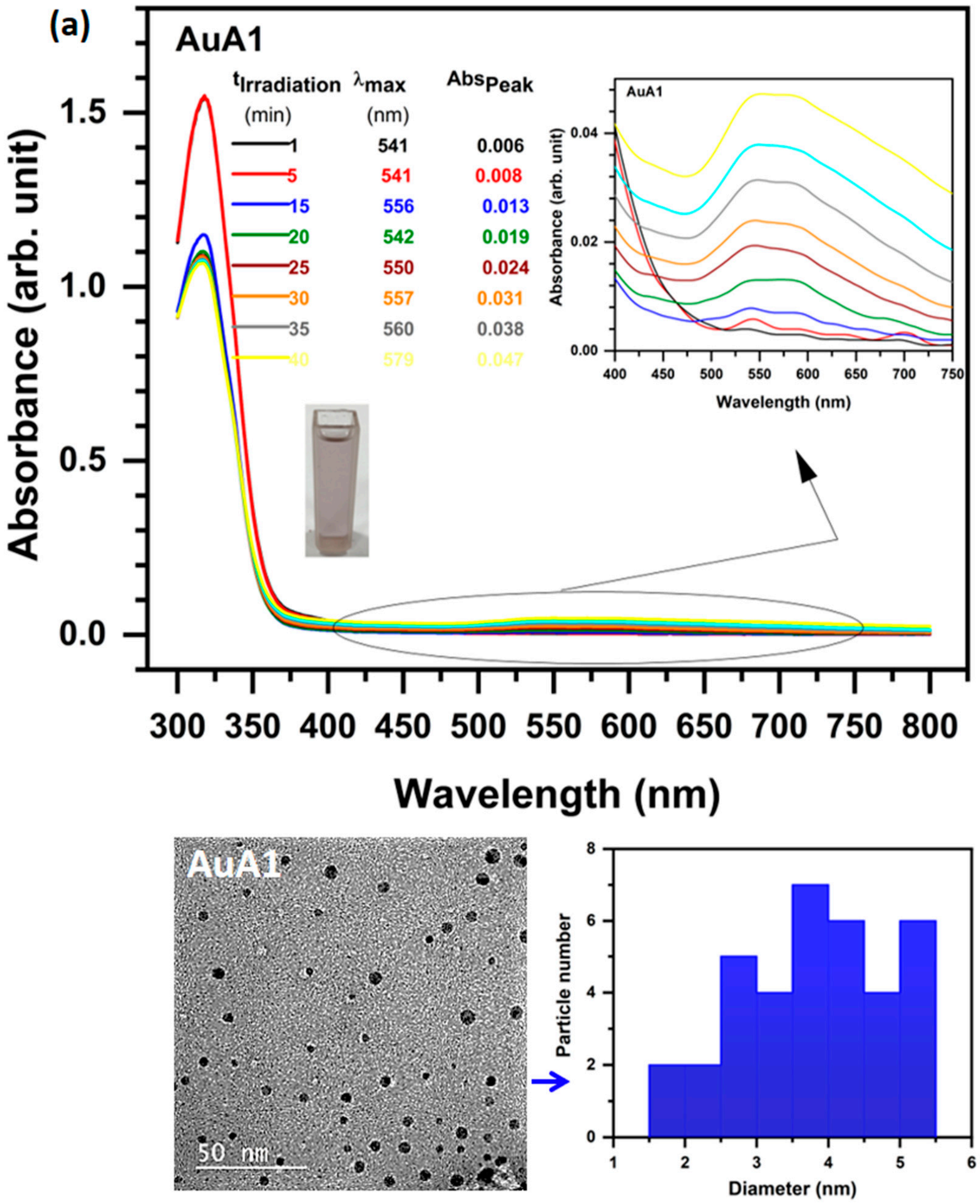

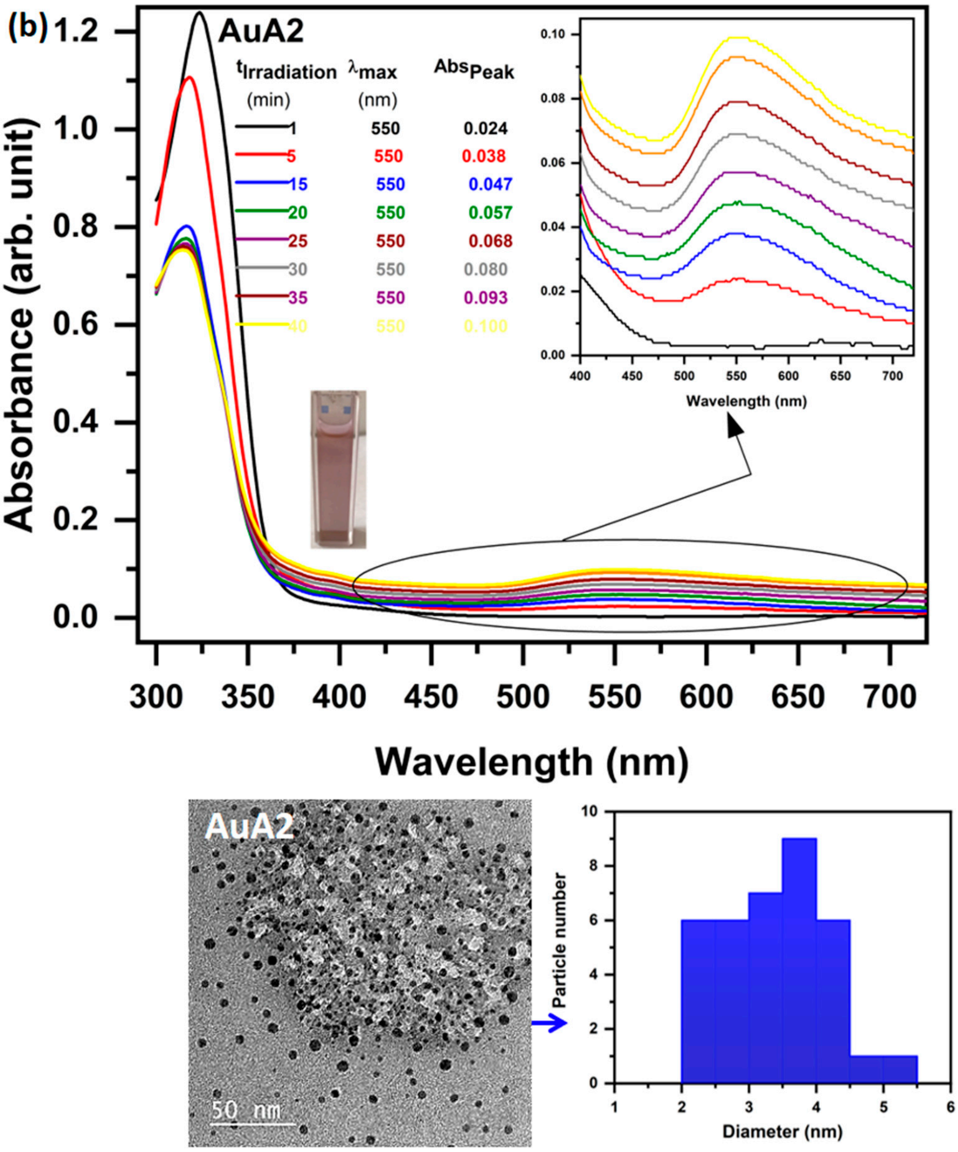

Figure S4. The absorption spectrum of gold nanoparticles obtained from photoreduction of $HAuCl_4$ with photoinitiator **A** at different concentrations; **(a)** AuA1 at 1wt% and (**b**) AuA2 at 3wt%. Corresponding TEM images of AuNPs with their respective size distributions.

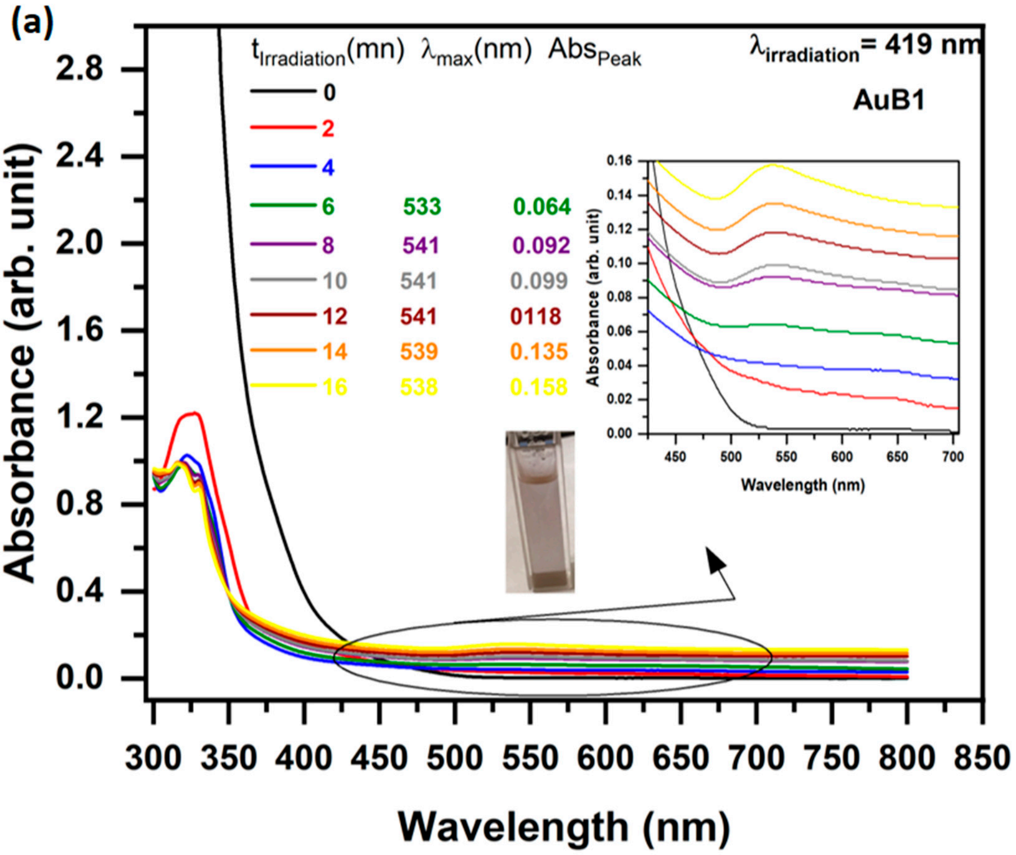
(a)
$t_{Irradiation}$(mn) λ_{max}(nm) Abs_{Peak}
$\lambda_{irradiation}$= 419 nm
AuB1
0
2
4
6 533 0.064
8 541 0.092
10 541 0.099
12 541 0118
14 539 0.135
16 538 0.158
Absorbance (arb. unit)
Wavelength (nm)
2.8
2.4
2.0
1.6
1.2
0.8
0.4
0.0
300 350 400 450 500 550 600 650 700 750 800 850
0.16
0.14
0.12
0.10
0.08
0.06
0.04
0.02
0.00
450 500 550 600 650 700

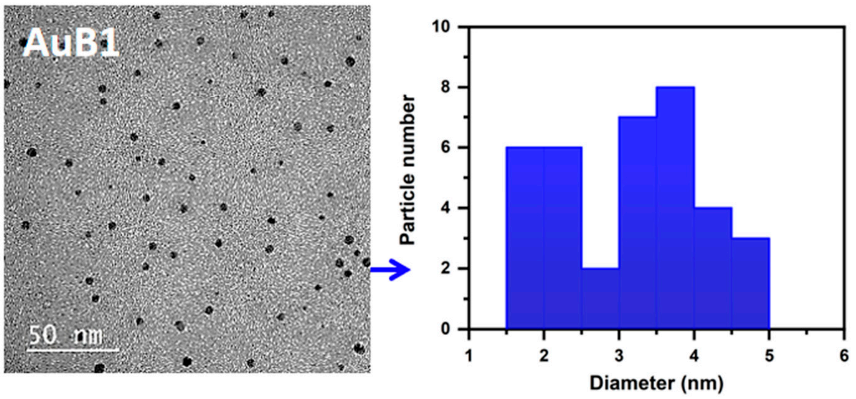
AuB1
50 nm
Particle number
Diameter (nm)
10
8
6
4
2
0
1 2 3 4 5 6

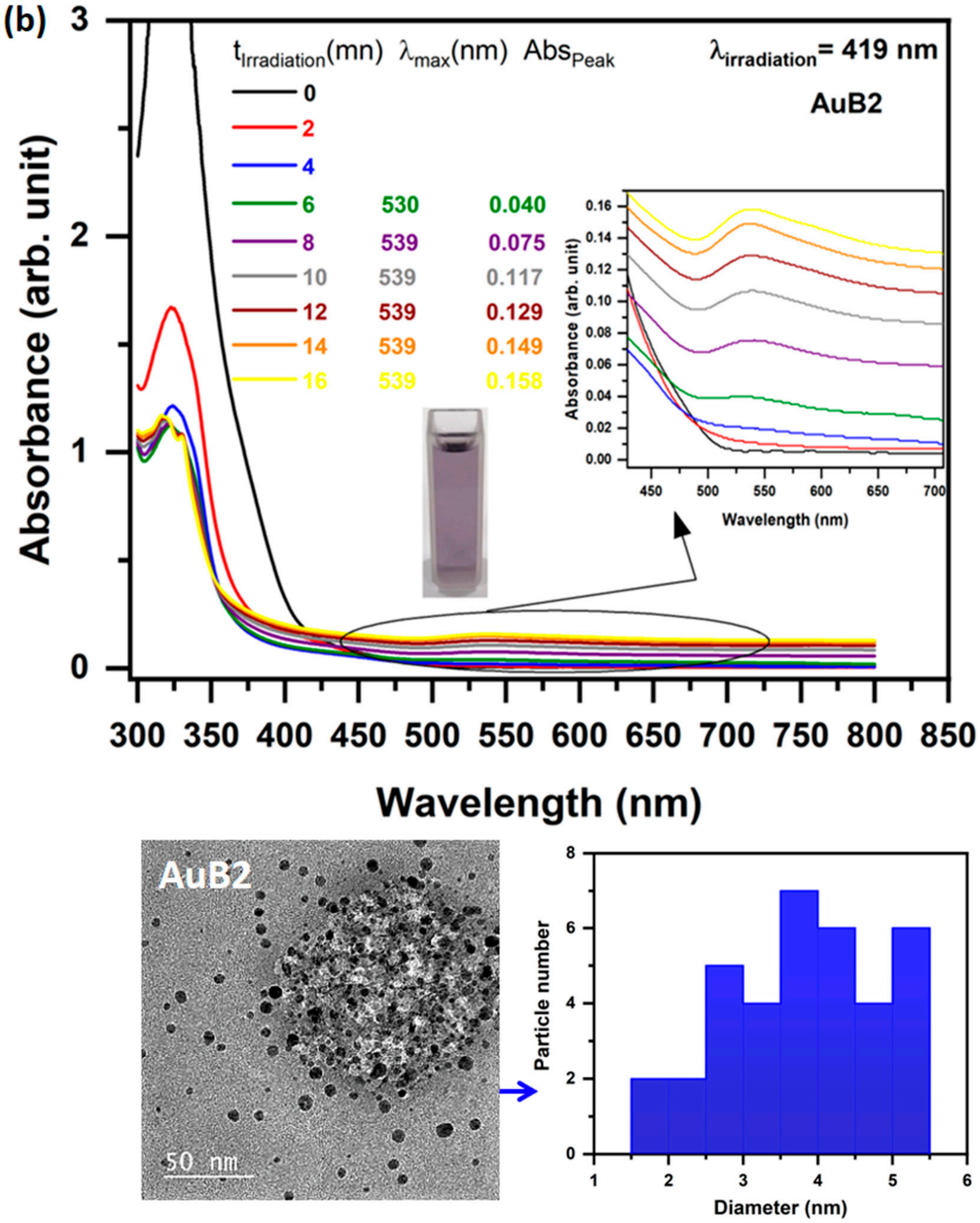

Figure S5. The absorption spectrum of gold nanoparticles obtained from photoreduction of $HAuCl_4$ with photoinitiator **B** at different concentrations; **(a)** AuB1 at 1wt% and (**b)** AuB2 at 3wt%. Corresponding TEM images of AuNPs with their respective size distributions.

4. *Photoproduction of Ag Nanoparticles*

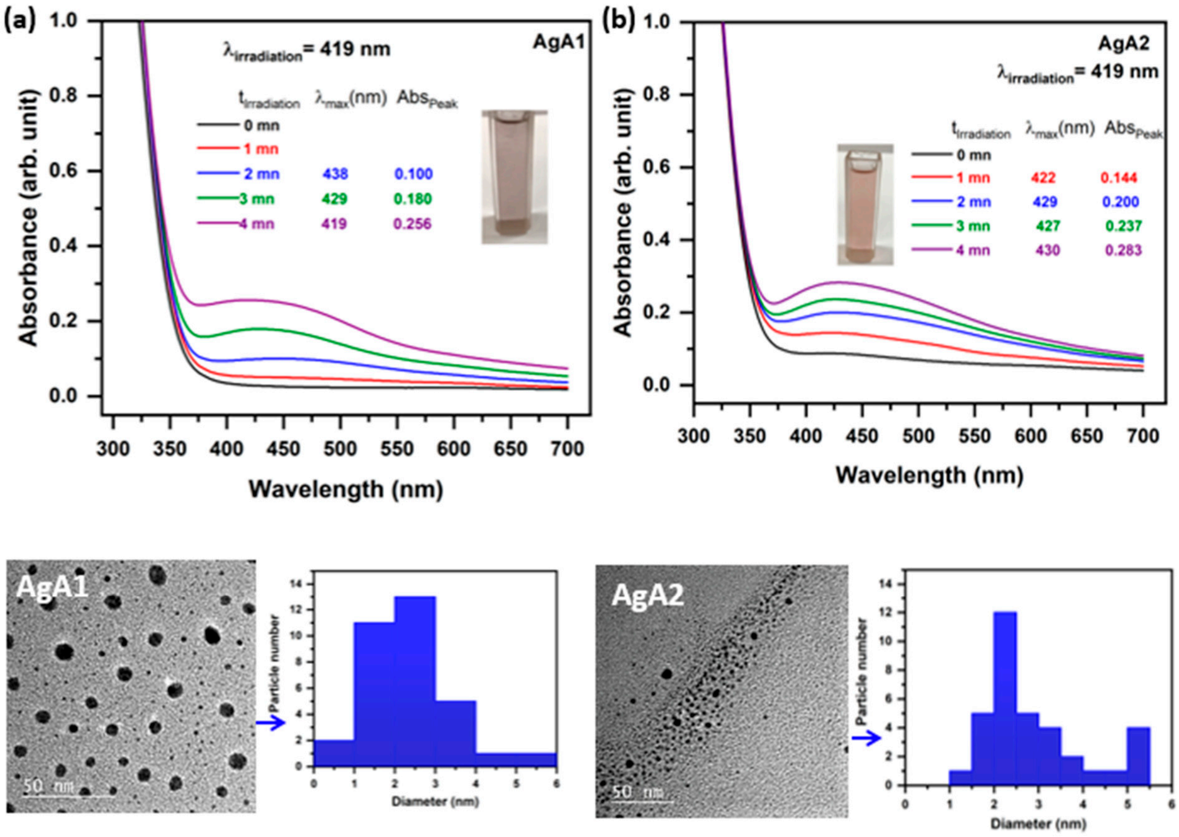

Figure S6. The absorption spectrum of silver nanoparticles obtained from photoreduction of $AgNO_3$ with photoinitiator **A** at different concentrations; **(a)** AgA1 at 1wt% and (**b)** AgA2 at 3wt%. Corresponding TEM images of AgNPs with their respective size distributions.

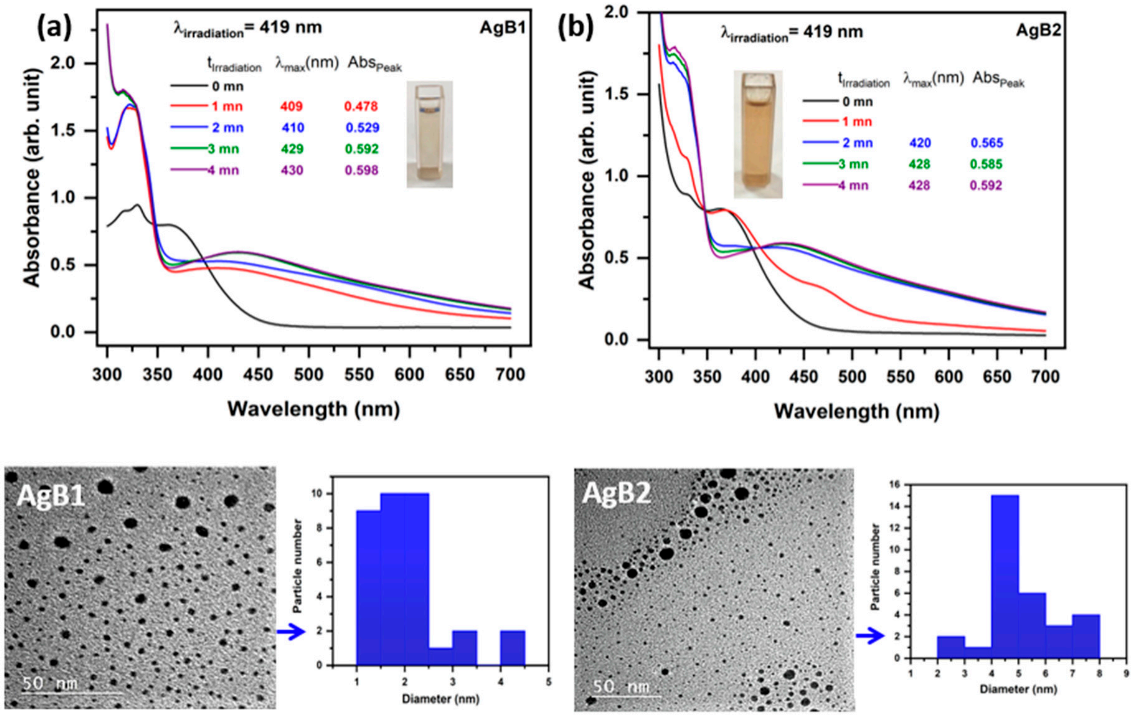

Figure S7. The absorption spectrum of silver nanoparticles obtained from photoreduction of $AgNO_3$ with photoinitiator **B** at different concentrations; **(a)** AgB1 at 1wt% and (**b)** AgB2 at 3wt%. Corresponding TEM images of AgNPs with their respective size distributions.

References

1. Ferguson, M.; Giri, N.; Huang, X.; Apperley, D.; James, S.L. One-Pot Two-Step Mechanochemical Synthesis: Ligand and Complex Preparation without Isolating Intermediates. *Green Chem.* **2014**, *16*, 1374–1382.
